# Body surface distribution of T wave alternans is modulated by heart rate and ventricular activation sequence in patients with cardiomyopathy

**DOI:** 10.1371/journal.pone.0214729

**Published:** 2019-04-10

**Authors:** Behnaz Ghoraani, Adrian M. Suszko, Raja J. Selvaraj, Anandaraja Subramanian, Sridhar Krishnan, Vijay S. Chauhan

**Affiliations:** 1 Department of Electrical and Computer Engineering, Ryerson University, Toronto, Canada; 2 Department of Computer and Electrical Engineering, Florida Atlantic University, Boca Raton, Florida, United States of America; 3 Division of Cardiology, University Health Network, Toronto, Canada; University of Minnesota, UNITED STATES

## Abstract

**Background:**

T wave alternans (TWA) is an electrocardiographic marker of heightened sudden death risk from ventricular tachyarrhythmias in patients with cardiomyopathy. TWA is evaluated from the 12-lead electrocardiogram, Frank lead, or Holter lead recordings, however these clinical lead configurations will not record TWA from adjacent regions of the body torso.

**Objective:**

We tested the hypothesis that changing heart rate or ventricular activation may alter the body surface distribution of TWA such that the clinical ECG leads fail to detect TWA in some patients; thereby producing a false-negative test.

**Methods:**

In 28 cardiomyopathy patients (left ventricular ejection fraction 28±6%), 114 unipolar electrograms were recorded across the body torso during incremental atrial pacing, followed by atrioventricular pacing at 100, 110 and 120bpm. TWA was measured from each unipolar electrogram using the spectral method. A clinically positive TWA test was defined as TWA magnitude (V_alt_) ≥1.9 uV with k ≥3 at ≤110bpm.

**Results:**

Maximum V_alt_ (TWA_max_) was greater from the body torso than clinical leads during atrial (p<0.005) and atrioventricular pacing (p<0.005). TWA_max_ was most prevalent in the right lower chest with atrial pacing 100 bpm and shifted to the left lower chest at 120 bpm. TWA_max_ was most prevalent in left lower chest with atrioventricular pacing at 100 bpm and shifted to the left upper chest at 120 bpm. Using the body torso as a gold standard, the false-negative rate for clinically positive TWA with clinical leads was 21% during atrial and 11% during atrioventricular pacing. Due to TWA signal migration outside the clinical leads, clinically positive TWA became false-negative when pacing mode was switched (atrial→atrioventricular pacing) in 21% of patients.

**Conclusions:**

The body surface distribution of TWA is modulated by heart rate and the sequence of ventricular activation in patients with cardiomyopathy, which can give rise to modest false-negative TWA signal detection using standard clinical leads.

## Introduction

Body surface microvolt T wave alternans (TWA) arises from beat-to-beat action potential alternans, and can presage ventricular tachyarrhythmias by increasing repolarization dispersion with subsequent conduction block and re-entry [1]. In patients with moderate to severe left ventricular dysfunction, TWA magnitude measured from Holter recordings and implantable cardioverter defibrillator (ICD) electrograms increases before ventricular tachyarrhythmias [2,3]. Several prospective clinical studies have shown a high negative predictive value (~97%/year) for sudden cardiac death with TWA testing in patients with cardiomyopathy [4] , while other studies [5,6] found no difference in adverse events between positive vs. negative TWA patients, and a lower negative predictive accuracy of 93%/year.

In these clinical studies, TWA testing was performed during exercise or pacing to increase heart rates up to 110 bpm, and TWA was evaluated from a localized region of the body torso transcribed by the 12-lead ECG and Frank lead configuration [7,8]. Using 114-electrode body surface potential mapping during atrial pacing at 110 bpm, we have previously shown that TWA consistently localized to the precordium, but that TWA underdetection may arise using the 12-lead ECG/Frank leads or Holter lead recording configurations [9]. Ventricular pacing has also been used in TWA testing when heart rates of 110 bpm cannot be achieved otherwise; however, discordant test results occur in up to 43% of patients between atrial and ventricular pacing [10–14]. The effect of heart rate and ventricular activation on the body surface distribution of TWA has not been studied, which is relevant to improving the accuracy of TWA testing and potentially its prognostic utility. We hypothesized that changes in heart rate or ventricular activation may alter the body surface distribution of TWA in some patients, such that the standard clinical lead configurations fail to detect TWA; thereby producing a false negative test.

Our objective was to compare changes in TWA magnitude across the entire body torso of patients with cardiomyopathy in response to incremental atrial and atrioventricular pacing. We also determined the prevalence of false negative TWA tests, whereby the TWA signal was not detectable using the clinical leads, but was still present in the remaining body torso.

## Materials and methods

### Patient population

Consecutive male patients with LV ejection fraction (LVEF) <40%, who either had a prophylactic dual-chamber ICD or were undergoing a clinical electrophysiology study, were prospectively included. The electrophysiology study was performed in the postabsorptive state to evaluate sudden cardiac death risk. Beta-blockers were not withheld prior to TWA evaluation or electrophysiology testing. Patients with atrial fibrillation, recent myocardial infarction (<1 month), unstable angina, New York Heart Association class 4 function, amiodarone use within 3 months, or pacemaker dependency were excluded. All patients gave written informed consent, and the study was approved by the research ethics boards at Mount Sinai Hospital, Toronto, and University Health Network, Toronto.

### Body torso mapping during pacing

After careful skin preparation, 114-electrodes (Biosemi, Amsterdam, Netherlands) were applied across the anterior and posterior thorax, and coincided with the standard position of the clinical 12-lead ECG, Frank leads, and Holter leads. Unipolar electrograms referenced to the Wilson central terminal were recorded unfiltered from each electrode at a sampling rate of 1,024 Hz and 24-bit dynamic range (31 nV resolution) using the Biosemi data acquisition system (Biosemi Inc, Amsterdam, Netherlands).

Pacing was performed while supine using either the ICD leads, or pacing catheters placed via the femoral vein for the clinical electrophysiology study. Atrial pacing at 100, 110 and 120 bpm was performed for 5 minutes at each rate using either the ICD atrial lead, or a quadripolar catheter (Avail™, Biosense Webster Inc.) in the high right atrium. Following atrial pacing, a 5-minute recontrol period without pacing was instituted. Thereafter, atrioventricular pacing (AV delay 160 ms) at 100, 110 and 120 bpm was performed for 5 minutes at each rate using the ICD pacing leads, or two quadripolar catheters (Avail™, Biosense Webster Inc.) placed in the high right atrium and right ventricular apex. Programmed pacing was achieved either through the ICD programmer, or a biostimulator (EP Medical Inc.).

### Clinical risk assessment using TWA

Among those patients undergoing clinical electrophysiology study, the body torso electrodes were removed following the above pacing protocol. Hi Res electrodes™ (Cambridge Heart Inc.) were then applied in the standard 12-lead ECG/Frank lead configuration [7]. Atrial pacing was performed with the quadripolar pacing catheter (Avail, Biosense Webster Inc.) at 100, 110 and 120 bpm for 5 minutes each. For the purpose of clinical risk stratification, the TWA evaluation during atrial or atrioventricular pacing was classified as either clinically positive (+), negative (-), or indeterminate using the automated Heartwave II algorithm (Cambridge Heart Inc.). A *clinically +TWA* test required TWA magnitude (V_alt_) ≥1.9uV, k ≥3, alternans duration >1 minute at heart rate ≤110bpm. Test results below this level, but still with k ≥3, were considered *clinically negative*. If neither a positive or negative test could be ascertained due to k <3, the test was classified as indeterminate. The k value reflects the signal-to-noise ratio and provides a measure of the reliability of TWA measurement. This clinical definition is associated with an increased risk of ventricular tachyarrhythmias and sudden death in patients with reduced ventricular function [4,7,8].

### Quantifying TWA from body torso mapping

For each patient, the TWA magnitude (V_alt_) and k value were determined for each unipolar electrode during pacing using the spectral method, as previously described by our group [9]. The largest V_alt_ in a 128-beat window (incremented by 16 beats) during the last 3 minutes of pacing was chosen to represent the TWA magnitude for each electrode. For each window, power spectra were computed at each time point in the JT segment and summated to create an aggregate power spectrum. The corresponding noise level in each window was computed as the mean spectral amplitude between 0.44 and 0.49 cycles per beat (cpb).

$$V_{alt}=\sqrt{power_{0.5}-mean\left(power_{0.44-0.49}\right)}$$

$$k_{value}=\frac{\left(power_{0.5}-mean\left(power_{0.44-0.49}\right)\right)}{\sigma \left(power_{0.44-0.49}\right)}$$

TWA_max_ was defined as *the* maximum V_alt_ (with k ≥3) recorded from the 114 body torso electrodes. For each patient, *detectable TWA* was defined as TWA magnitude (V_alt_) >0uV with k ≥3 in >1 unipolar lead for any duration and at any pacing rate. In contrast, *positive (+) TWA* was defined as V_alt_ ≥1.9uV with k ≥3 in >1 unipolar lead for any duration and at any pacing rate. If these conditions were not satisfied, TWA was considered *negative (-)*. An *indeterminate TWA* classification was also considered if k<3 for each 128-beat window of the 3-minute recording.

### Clinical leads and clinically positive TWA

The clinical leads were derived from the 114-body torso electrodes, and included (i) standard 12-leads (I, II, III, aVL, aVF, aVR, V1-V6), (ii) vector magnitude lead (V_m_), and (iii) standard bipolar Holter leads (CM1, CM3, CM5). V_m_ was computed from the orthogonal Frank leads X, Y, Z as previously described [8,15]. The bipolar ECG leads and Holter leads were constructed by subtracting the potentials recorded from their constituent unipolar electrodes [16]. For the body torso lead set, *clinically positive (+) TWA* was defined as V_alt_ ≥1.9uV with k ≥3 in more than 1 unipolar lead at pacing rates ≤110 bpm. For the clinical leads, *clinically +TWA* was defined as V_alt_ ≤1.9uV with k ≤3 in more than 1 standard 12-lead, V_m_ alone, or ≤1 Holter lead at pacing rates ≤110 bpm [7,8]. If these conditions were not satisfied, TWA test was considered *clinically negative*. An *indeterminate TWA* classification was also considered if k<3 for each 128-beat window of the 3-minute recording.

### Statistical analysis

Continuous variables are expressed as mean and standard deviation or median and interquartile range (25^th^-75^th^ percentiles) where appropriate. The Wilcoxon signed-rank test was used for paired comparison between groups. Categorical variables are presented as frequency or percentage. A two-tailed *p<*0.05 was considered statistically significant. All statistical analysis was performed using SPSS (version 19.0.0, SPSS Inc.).

## Results

### Patient population

The study population consisted of 28 male patients (63±10 years, LVEF 28±6%) with ischemic (n = 19, 68%) or nonischemic cardiomyopathy (n = 9, 32%). Their clinical characteristics are summaries in Table 1.

**Table 1:** Patient characteristics.

Age (years)		63 ± 10
Male, n (%)		28 (100)
LV ejection fraction		28 ± 6%
QRS duration (ms)		133 ± 38
Cardiomyopathy		
	Ischemic, n (%)	19 (68)
	Nonischemic, n (%)	9 (32)
History of sustained ventricular tachyarrhythmia, n		0
Medications		
	Beta-blocker, n (%)	28 (100)
	Class III antiarrhythmic (sotalol, amiodarone), n	0

### Effect of body torso sampling and pacing mode on detectible TWA and +TWA

In 21 patients, body torso mapping of TWA using 114-electrodes was completed during atrial pacing at 100, 110 and 120 bpm. In 7 patients, atrial pacing could not be completed at the three prespecified pacing rates due to AV nodal Wenckebach at 110 or 120 bpm. All 21 (100%) patients had detectible TWA (V_alt_ >0, k ≥3) in more than 1 unipolar lead. However, +TWA (V_alt_ ≥1.9uV, k ≥3) was present in 18 (86%) patients. There were no indeterminate TWA results. When only the clinical leads were considered 18 (86%) patients still had detectible TWA, but only 16 (76%) patients had +TWA.

In 28 patients, body torso mapping of TWA using 114-electrodes (including clinical leads) was completed during atrioventricular pacing at 100, 110 and 120 bpm. All 28 (100%) patients had detectible TWA as well as +TWA. When only the clinical leads were analyzed, 27 (96%) patients had detectible TWA and +TWA. There were no indeterminate TWA results.

### Effect of body torso sampling, pacing rate, and pacing mode on TWA_max_

**Fig 1:**
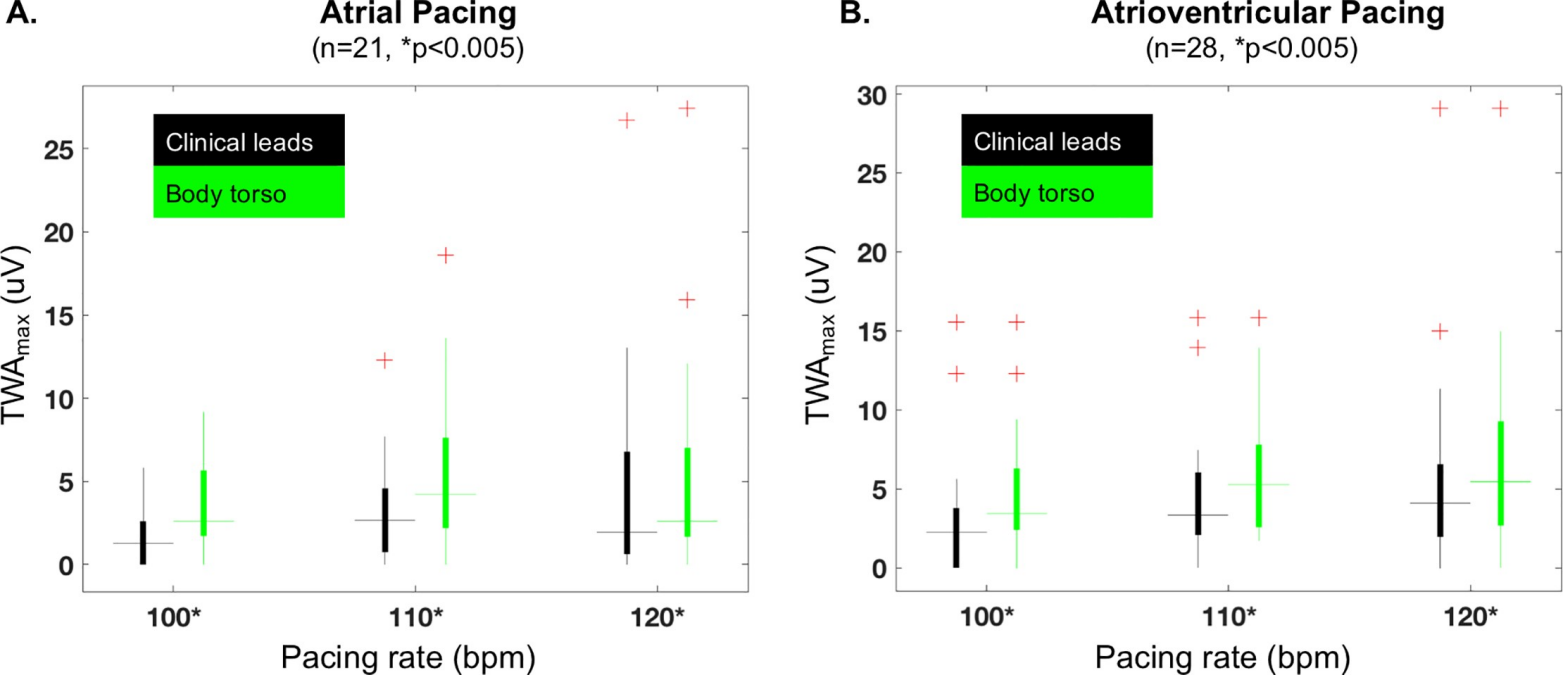
TWA_**max**_ is compared between clinical leads and body torso for atrial (**A**) and atrioventricular pacing (**B**). TWA_**max**_ is consistently greater when body torso is sampled vs. clinical leads, regardless of the pacing mode or rate. + indicates outliers.

**Fig 2:**
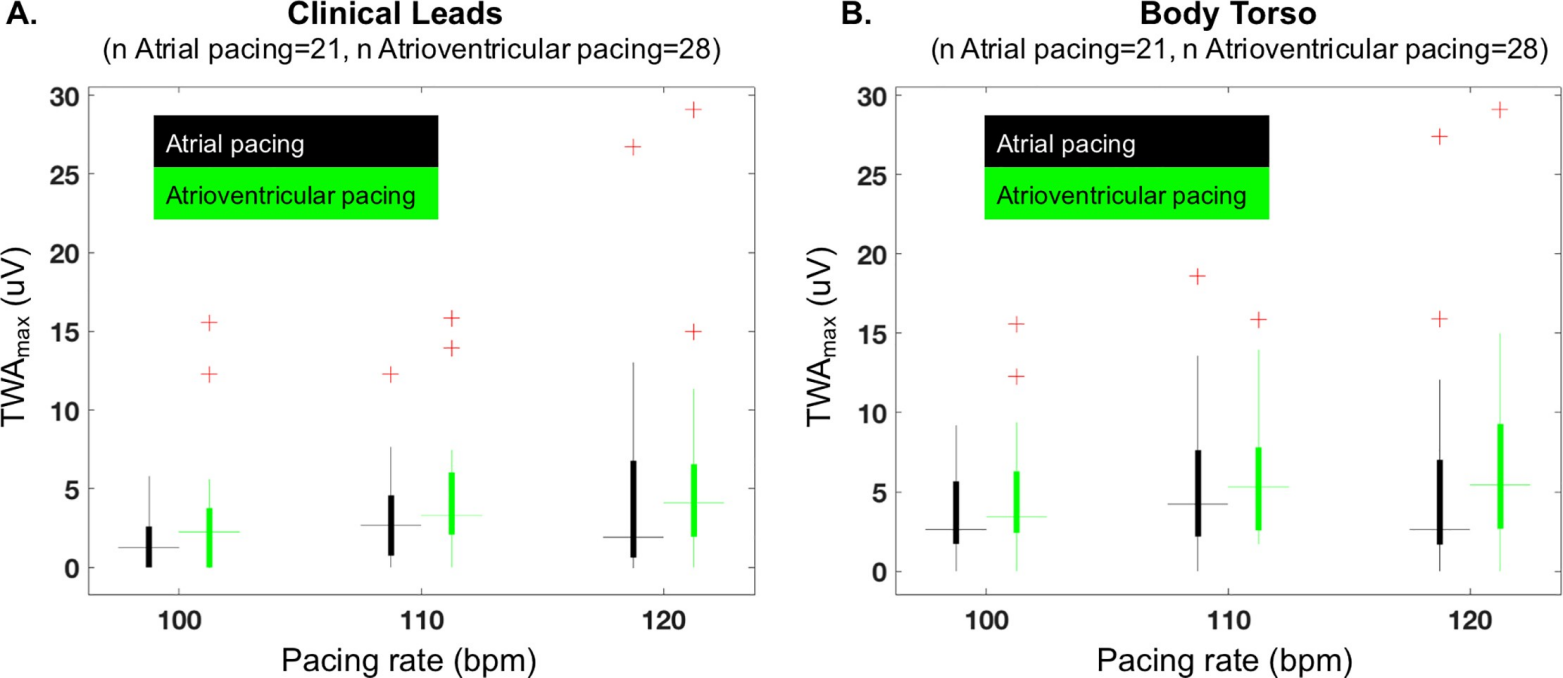
TWA_**max**_ is compared between atrial and atrioventricular pacing for clinical leads (**A**) and body torso (**B**). TWA_**max**_ is similar between atrial and atrioventricular pacing, regardless of clinical leads or body torso sampling. + indicates outliers.

Comparison of TWA_max_ (maximum V_alt_, k ≥3) from the body torso vs. clinical leads is shown in Fig 1. During atrial (Fig 1A) and atrioventricular pacing (Fig 1B), TWA_max_ was greater when measured from the body torso vs. clinical leads (p<0.005). Fig 2 compares the effect of atrial vs. atrioventricular pacing on TWA_max_. There was no difference in TWA_max_ with the mode of pacing regardless of the pacing rate or the recording configuration.

### Effect of pacing rate and pacing mode on TWA_max_ body surface distribution

**Fig 3:**
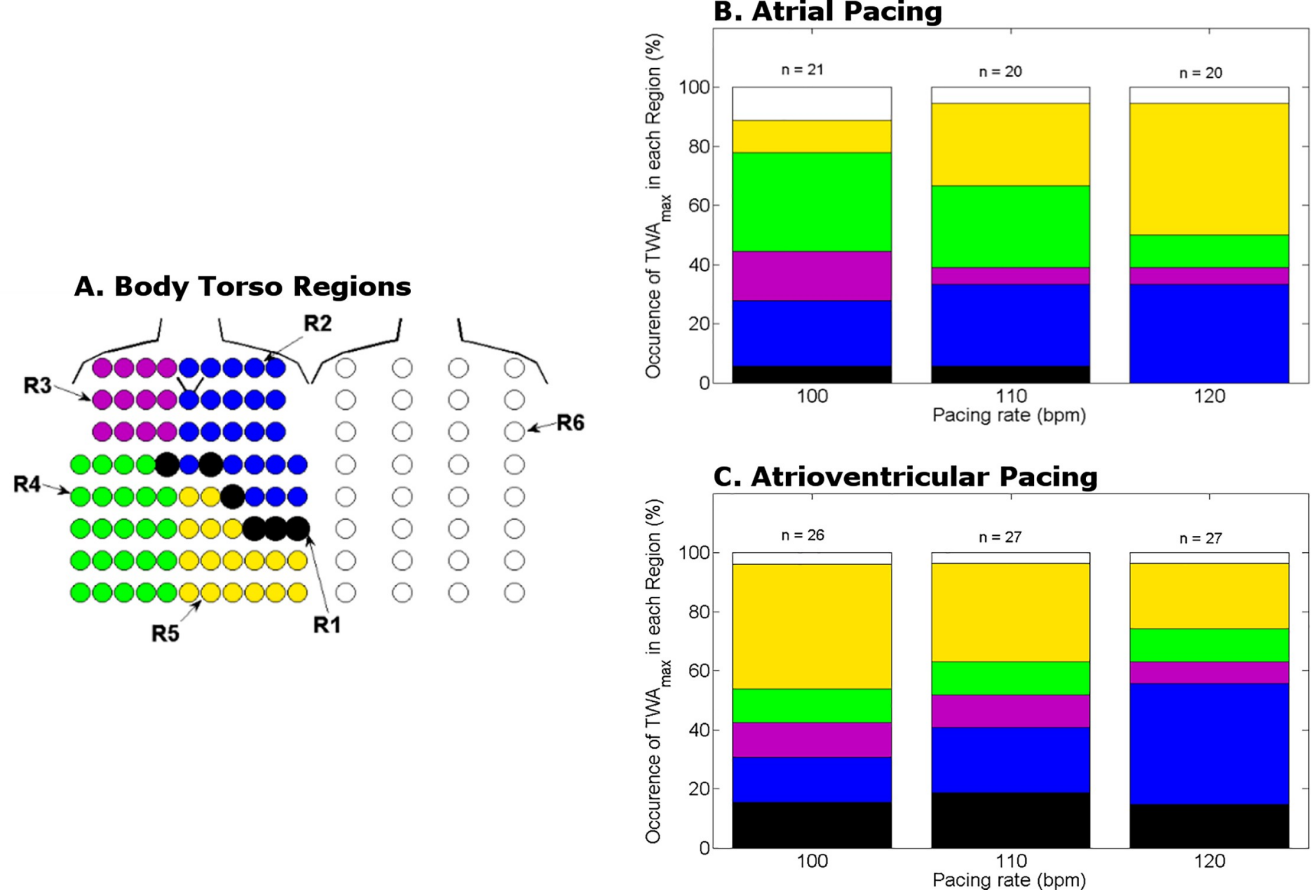
Body torso distribution of TWA_max_ during atrial and atrioventricular pacing. The body torso was divided into 6 regions (**A**). R1 indicates the location of the standard precordial leads, V1-V6. R6 is the posterior chest. With increasing atrial pacing rate, TWA_**max**_ shifted from R4 to R5 (**B**). With increasing atrioventricular pacing rate, TWA_**max**_ relocated from R5 to R2 (**C**).

The body torso was divided into 6 regions as shown in Fig 3A with 5 regions located anteriorly and 1 posteriorly. The prevalence of TWA_max_ in each region was determined during incremental atrial (Fig 3B), then atrioventricular pacing (Fig 3C). The precordial leads, V1-V6, were represented by Region 1, where TWA_max_ was located in ≤18% of patients whether during atrial or atrioventricular pacing. TWA_max_ was most prevalent in the right lower chest (Region 4) with atrial pacing at 100 bpm, but then shifted to the left lower chest (Region 5) with higher pacing rates. With atrioventricular pacing at 100 bpm, TWA_max_ was most prevalent in Region 5 and redistributed to the left upper chest (Region 2) at higher pacing rates.

We also performed per patient analysis of TWA_max_ localization with changing heart rate. The prevalence of 3 different responses during increasing pacing rate was determined as follows, (i) no change in TWA_max_ distribution, (ii) transient shift in TWA_max_ distribution at 110 and 120 bpm with return to the baseline distribution at 100 bpm, and (iii) complete shift in TWA_max_ distribution at 110 and 120 bpm compared to the baseline distribution at 100bpm. With atrial pacing, the prevalence of these 3 groups was 5%, 57% and 38%, respectively; while with atrioventricular pacing, the prevalence was 11%, 43% and 46%, respectively. Therefore, it was unusual for TWA_max_ to remain in the same location for a given patient but shifting to adjacent locations was more common.

### Effect of body surface sampling, pacing rate and pacing mode on clinically +TWA

Clinically +TWA (V_alt_ ≥1.9uV, k ≥3, rate ≤110bpm) based on the proprietary algorithm (Heartwave II™, Cambridge Heart Inc.) was present in 15 (71%) of 21 patients. These same patients also had clinically +TWA (V_alt_ ≥1.9uV, k ≥3, rate ≤110bpm) as defined by our algorithm using the clinical leads, thereby supporting the validity of our algorithm. There were no indeterminate TWA results using the proprietary algorithm.

**Fig 4:**
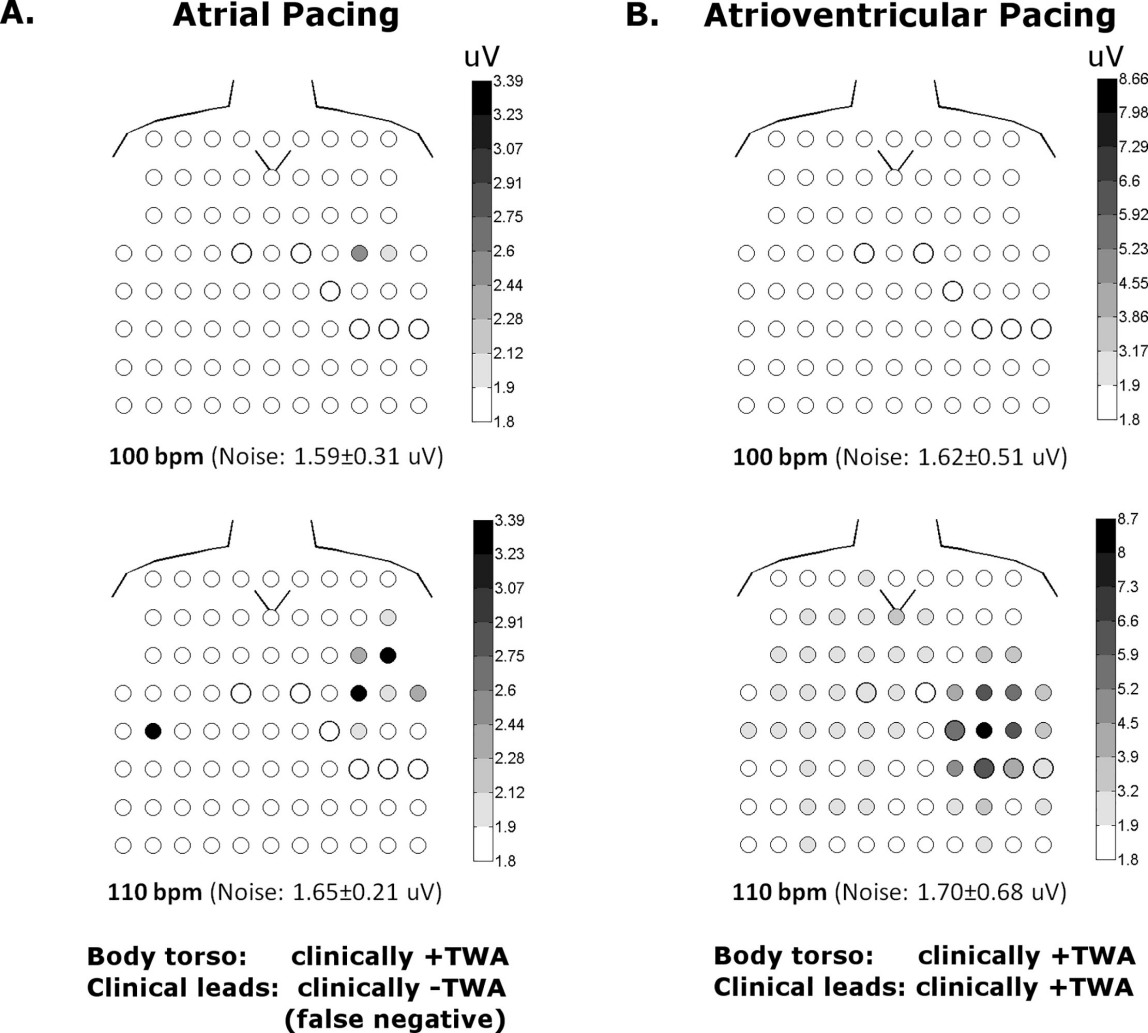
Body torso maps of V_alt_ (uV) in one patient during atrial and atrioventricular pacing. The anterior torso with V1-V6 is indicated by the bold circles and the V symbol depicts the sternal notch. The posterior torso is not shown for simplicity as there is virtually no TWA signal. With atrial pacing (**A**), TWA is detected outside the clinical leads at 100 and 110 bpm. This patient would be classified as clinically–TWA using clinical leads, but +TWA with body torso mapping (i.e. false negative). With atrioventricular pacing (**B**), TWA is detected in body torso as well as clinical leads at 110, but not 100 bpm. Thus atrioventricular pacing would classify this patient as clinically +TWA using clinical leads, while atrial pacing resulted in a clinically negative test (i.e. false negative).

**Fig 5:**
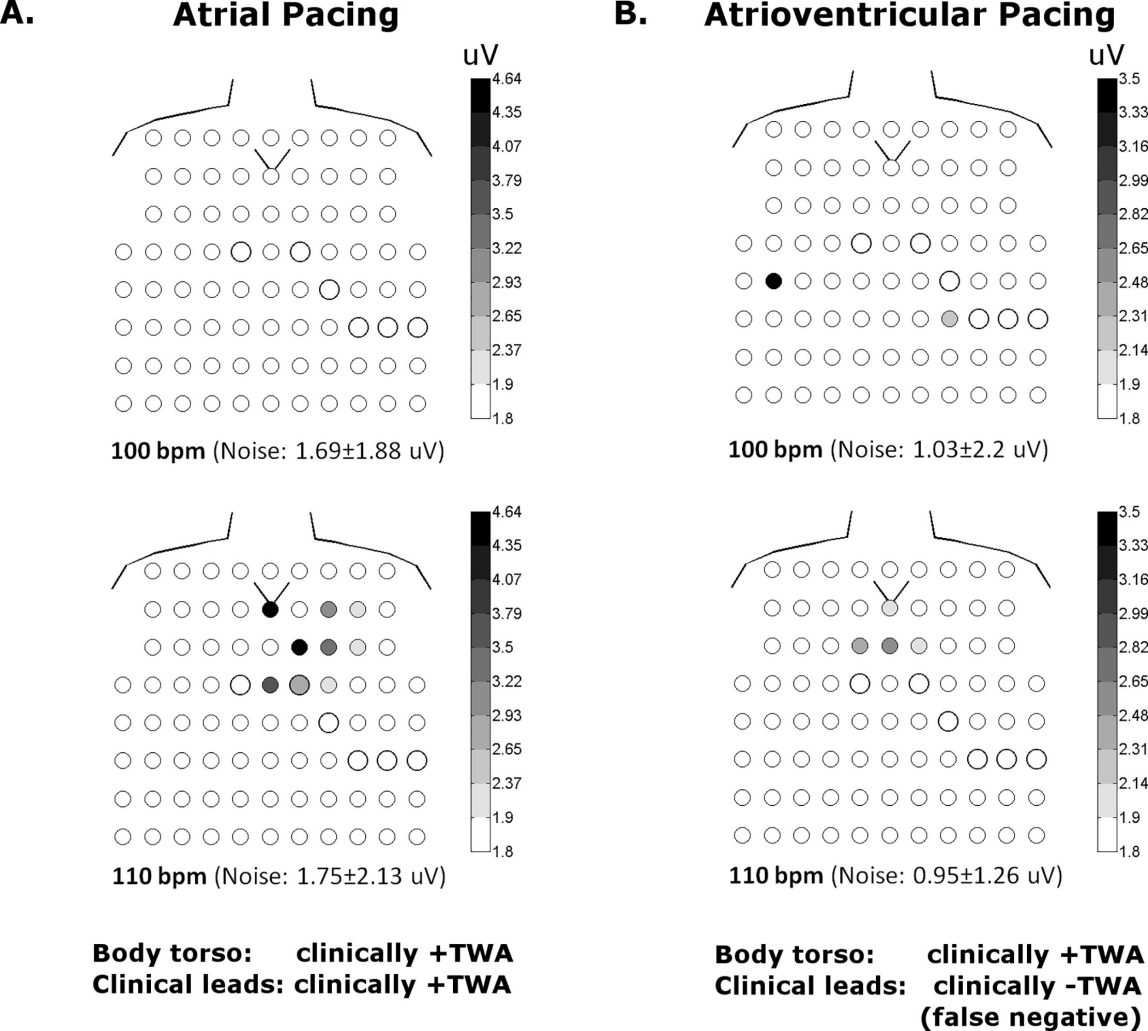
Body torso maps of V_alt_ (uV) in one patient during atrial and atrioventricular pacing. With atrial pacing (**A**), TWA is detected in body torso as well as clinical leads only at 110 bpm. With atrioventricular pacing (**B**), TWA is no longer detected in clinical leads, but remains detectible in body torso. Using clinical leads alone, this patient would be classified as clinically +TWA, but -TWA with atrioventricular pacing.

The effect of body surface sampling, either from the body torso or clinical leads, on the classification of clinically +TWA is shown in Tables 2 and 3. Among 21 patients, 6 (29%) were clinically negative during atrial pacing when only clinical leads were considered. In 4 (67%) of these 6 patients, TWA became clinically positive when measured from the entire body torso. This is illustrated for one patient in Fig 4A. With atrioventricular pacing, 4 (14%) of 28 patients were clinically negative when measured from the clinical leads, and 3 (75%) of these patients became clinically positive when the body torso was considered, as shown for one patient in Fig 5B. On the other hand, if clinically +TWA was detected by the clinical leads, the classification did not change when the body torso was assessed irrespective of atrial or atrioventricular pacing.

**Table 2:** Effect of lead configuration on clinically +TWA during atrial pacing.

Atrial Pacing n = 21		*Body Torso* *100 and 110 bpm*		Total
		+ TWA	- TWA	
*Clinical Leads* *100 and 110 bpm*	**+ TWA**	15 (100%)	0 (0%)	15 (100%)
	**- TWA**	4 (67%)	2 (33%)	6 (100%)

**Table 3:** Effect of lead configuration on clinically +TWA during atrioventricular pacing.

Atrioventricular Pacing n = 28		*Body Torso* *100 and 110 bpm*		Total
		+ TWA	- TWA	
*Clinical Leads* *100 and 110 bpm*	**+ TWA**	24 (100%)	0 (0%)	24 (100%)
	**- TWA**	3 (75%)	1 (25%)	4 (100%)

**Fig 6:**
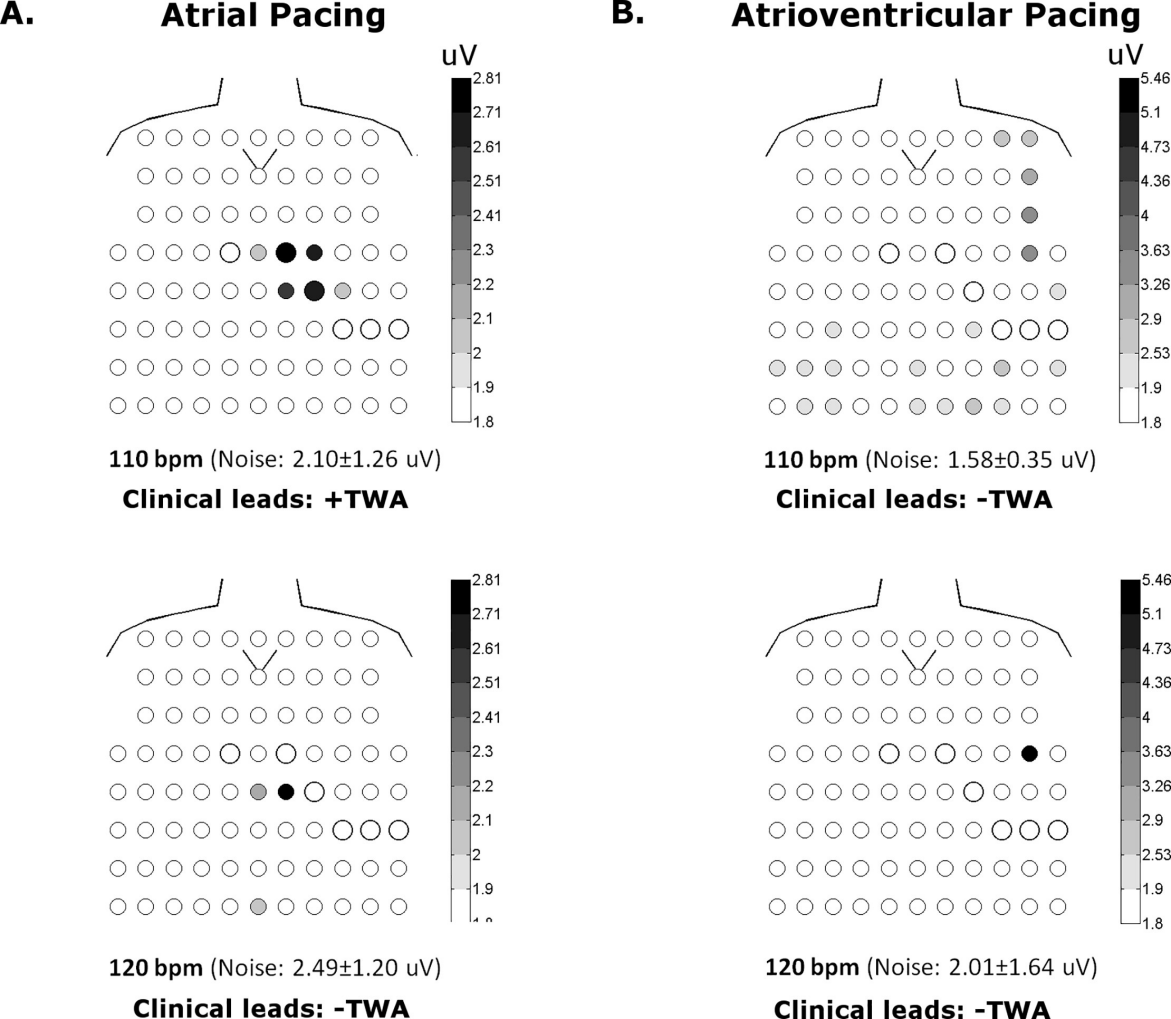
Body torso maps of V_alt_ (uV) in one patient during atrial and atrioventricular pacing. With atrial pacing (**A**), TWA is detected in body torso as well as clinical leads at 110 bpm. However, TWA is no longer detected in clinical leads at 120 bpm, but still evident in body torso leads below the clinical leads. Using clinical leads alone, the clinical TWA classification would still remain positive because pacing rates above 110 bpm are not considered. With atrioventricular pacing (**B**), TWA is no longer detectible in the clinical leads at 110 or 120 bpm, but still present in the body torso. Although this patient would still be classified as clinically +TWA based on atrial pacing 110 bpm alone, changes in pacing rate or mode have altered the body surface distribution of TWA.

**Fig 7:**
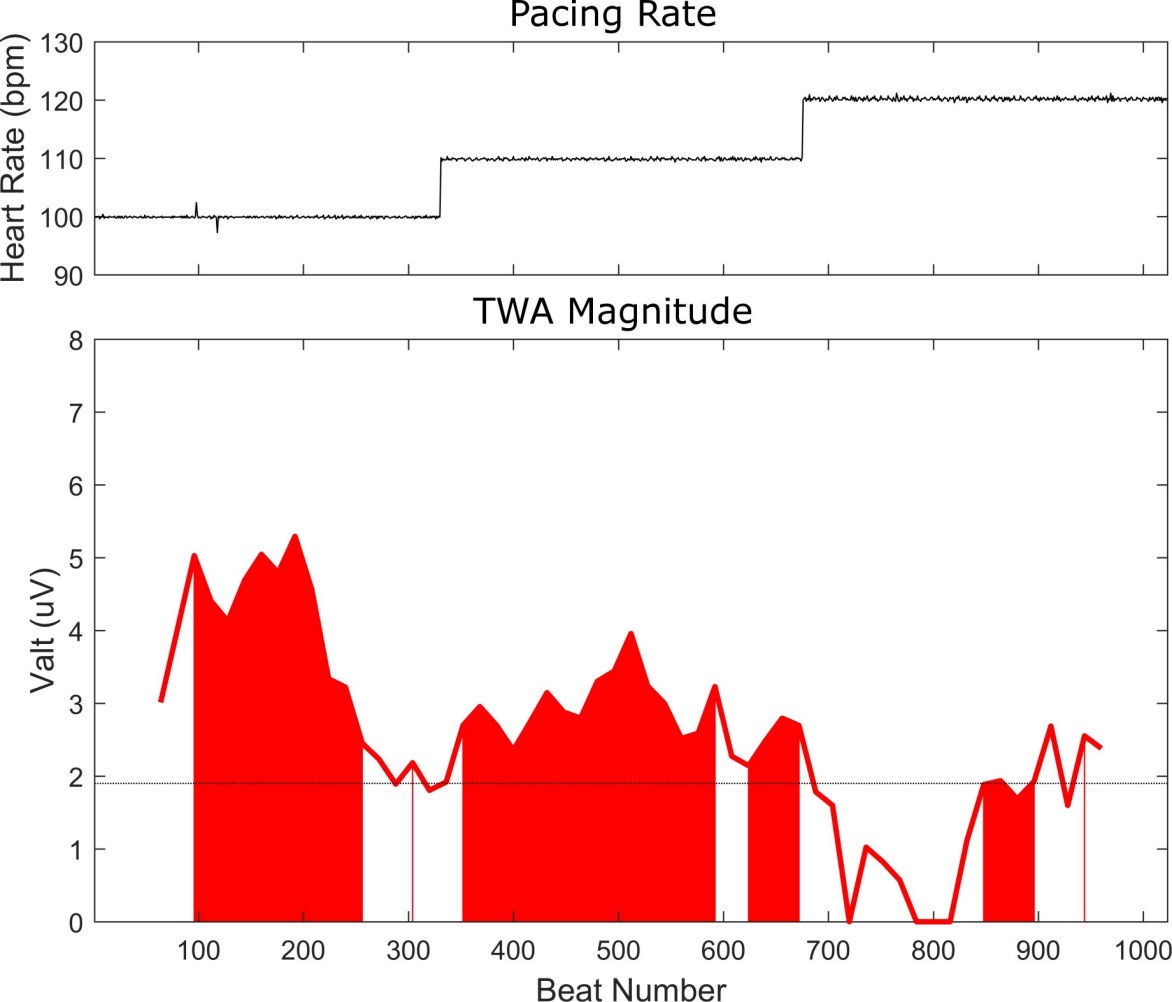
Illustration of TWA magnitude attenuation (lower panel) associated with increasing atrioventricular pacing rate (upper panel) in a precordial lead of a representative patient. The TWA magnitude decreases by ~1.5 uV with each subsequent 10 bpm increase in rate, causing TWA magnitude to fall below clinical significance at 120 bpm. The red shaded regions indicate V_alt_ values with a k ≥3. The dotted black line denotes the clinical TWA V_alt_ cutpoint of 1.9 uV.

**Fig 8:**
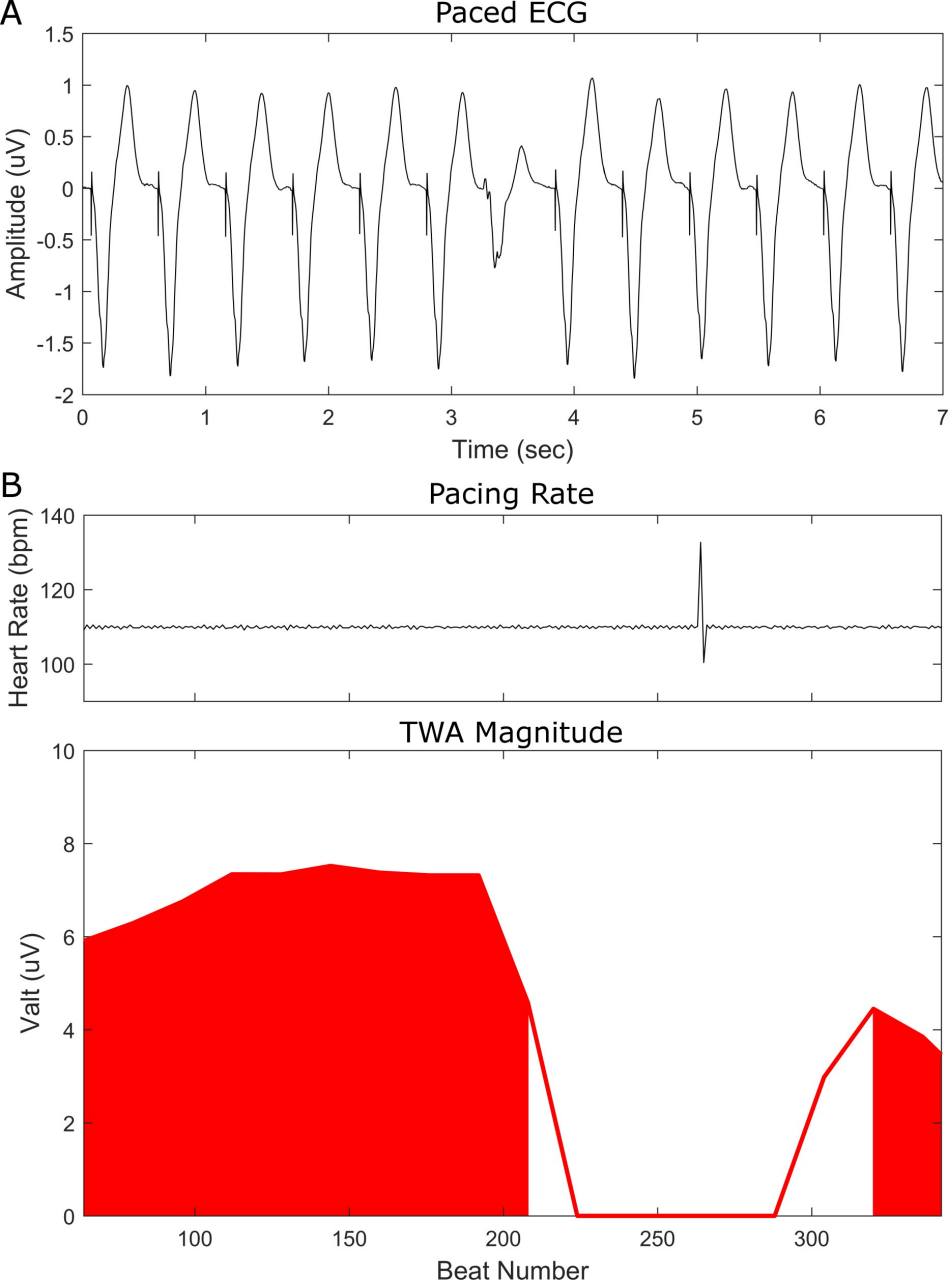
(A) ECG depicting a premature ventricular ectopic beat during atrioventricular pacing at 110 bpm in a precordial lead of a representative patient. (B) Illustration of TWA nullification (lower panel) associated with the region surrounding the ectopic beat (upper panel) and the attenuation of TWA magnitude post ectopic. The red shaded regions indicate V_alt_ values with a k ≥3.

The effect of pacing rate (100 or 110 bpm vs. 120 bpm) on the reclassification of TWA was also considered, while maintaining body surface sampling to the clinical leads (Tables 4 and 5). Among the 6 patients with clinically -TWA during atrial pacing at 100 or 110 bpm, 1 (17%) became positive at 120 bpm. In the case of atrioventricular pacing, 4 patients had clinically–TWA at 100 or 110 bpm, and 3 (75%) of these 4 became positive at 120 bpm. We also considered the effect of a higher pacing rate (100 or 110 bpm vs. 120 bpm) on the conversion of a clinically +TWA to–TWA, which occurred in 5 (33%) of 15 patients during atrial pacing at 120 bpm, and 6 (25%) of 24 patients during atrioventricular pacing at 120 bpm. This reclassification was due to redistribution of the TWA signal to the body torso regions R2 (n = 2), R4 (n = 2) and R5 (n = 2) remote from clinical leads in 6 (55%) patients, while in another 3 (27%) patients premature beats at the onset of the rate change were causal. Fig 6A is an example of a patient who had clinically +TWA during atrial pacing at 110 bpm which then became negative at 120 bpm due to relocation of the signal (V_alt_ 2.81uV) to the body torso region R5 outside the clinical leads. Fig 7 illustrates a patient with decreasing TWA magnitude measured in a precordial lead as a function of increasing atrioventricular pacing rate, such that clinically +TWA at 100 and 110bpm, then became–TWA at 120bpm. Fig 8 shows another patient with a reduction in TWA magnitude measured in a precordial lead during atrioventricular pacing at 110bpm after a premature ventricular beat.

**Table 4:** Effect of pacing rate on clinically +TWA during atrial pacing.

Atrial Pacing n = 21		*Clinical Leads* *120 bpm*		Total
		+ TWA	- TWA	
*Clinical Leads* *100 and 110 bpm*	**+ TWA**	10 (67%)	5 (33%)	15 (100%)
	**- TWA**	1 (17%)	5 (83%)	6 (100%)

**Table 5:** Effect of pacing rate on clinically +TWA during atrioventricular pacing.

Atrioventricular Pacing n = 28		*Clinical Leads* *120 bpm*		Total
		+ TWA	- TWA	
*Clinical Leads* *100 and 110 bpm*	**+ TWA**	18 (75%)	6 (25%)	24 (100%)
	**- TWA**	3 (75%)	1 (25%)	4 (100%)

Finally, the effect of pacing mode (atrial vs. atrioventricular pacing) on the reclassification of clinically +TWA was evaluated, again while maintaining body surface sampling to the clinical leads (Tables 6 and 7). Conversion of a clinically +TWA to–TWA occurred in 3 (20%) of 15 patients with atrial→atrioventricular pacing (Figs 5A, 5B, 6A and 6B) and 5 (28%) of 18 patients with atrioventricular→atrial pacing (Fig 4A and 4B). This reclassification was due to redistribution of TWA to regions remote from the clinical leads in 4 (50%) of these 8 patients.

**Table 6:** Effect of pacing mode on clinically +TWA beginning with atrial pacing.

n = 21		*Clinical Leads* Atrioventricular Pacing *100 and 110 bpm*		Total
		+ TWA	- TWA	
*Clinical Leads* **Atrial Pacing** *100 and 110 bpm*	**+ TWA**	12 (80%)	3 (20%)	15 (100%)
	**- TWA**	4 (67%)	2 (33%)	6 (100%)

**Table 7:** Effect of pacing mode on clinically +TWA beginning with atrioventricular pacing.

n = 21		*Clinical Leads* Atrial Pacing *100 and 110 bpm*		Total
		+ TWA	- TWA	
*Clinical Leads* **Atrioventricular Pacing** *100 and 110 bpm*	**+ TWA**	13 (72%)	5 (28%)	18 (100%)
	**- TWA**	2 (67%)	1 (33%)	3 (100%)

## Discussion

The major finding of our study is that the body surface distribution of TWA is modulated by heart rate and the sequence of ventricular activation. This is based on the following: (i) TWA_max_ is not prevalent over the precordial leads (i.e. V1-V6 leads), but localizes most often to the right lower and left lower chest with atrial and atrioventricular pacing, respectively, and (ii) the location of TWA_max_ shifts in a counterclockwise direction over the anterior chest with increasing pacing rates. As a consequence, the detection of TWA (V_alt_ >0, k ≥3) is influenced by pacing rate and mode when sampling is confined to the standard clinical leads rather than the entire body torso. In the case of TWA_max_, the clinical leads underestimate TWA magnitude compared to the body torso regardless of pacing rate or pacing mode.

The body surface projection of TWA is primarily dependent on the spatial and transmural location of alternating action potential sources in the ventricle as well as the rotation of the heart in the torso. We have previously shown that varying the action potential alternans source location in a simulated heart model can change the distribution of TWA on the body torso [9]. In patients undergoing body surface potential mapping during atrial pacing at 110 bpm, there is also considerable variation in the spatial distribution of TWA over the anterior thorax, although the greatest TWA magnitudes tend to localize over the precordial region [9]. The present study extends these finding by showing variations in TWA body surface mapping in the same patient as a function of increasing heart rate and changing ventricular activation from atrial to atrioventricular pacing.

In animal studies using Langendorff-perfused intact normal hearts [1,17,18] and arterially-perfused LV wedges from cardiomyopathic hearts, [19] faster heart rates increase optical action potential alternans magnitude, and convert spatially concordant action potential alternans to spatially discordant alternans. Although this coincides with an increase in the magnitude of TWA on a volume-averaged electrocardiogram, the effect on the body surface distribution of TWA has not been directly studied. Spatial heterogeneity in electrical alternans recorded from unipolar electrograms across the endocardium and epicardium has been described by our group in patients with cardiomyopathy [20]. Based on experimental studies, this heterogeneity likely arises from regional differences in intracellular calcium cycling as a result of nonuniform ion channel remodelling in the myopathic heart [21,22]. The presence of heterogeneous structural and functional barriers from anatomically remodelled infarcted myocardium with abnormal conduction velocity restitution can also produce regional action potential alternans and discordant alternans in proximity to the barrier. The direction of wave propagation and anisotropic conduction may further affect the spatial distribution of action potential alternans, particularly in relationship to a structural barrier [23,24]. Thus, increasing heart rate or altering ventricular activation in our myopathic patients may produce unique body surface TWA patterns by virtue of regional differences in action potential alternans. We speculate that the systematic counterclockwise shift in TWA_max_ across the anterior chest at higher pacing rates with either atrial or atrioventricular pacing may be the result of a greater mass of alternating myocardium.

### Clinical implications

Although a clinically +TWA test using the spectral method is based on a V_alt_ ≥1.9uV (k ≥3), higher TWA magnitude may have incremental prognostic utility. In patients with ischemic and nonischemic cardiomyopathy, Klingenheben et al [25] showed that arrhythmic events were associated with higher V_alt_ and a greater number of TWA positive ECG leads. In our study, less than 15% of patients had TWA_max_ localized to the precordial leads (ie. V1-V6) and TWA_max_ was lower when measured from the clinical leads compared to the body torso. Using the clinical leads alone, the false negative rate for clinical TWA testing was 21% with atrial pacing and 11% with atrioventricular pacing based on the body torso as the gold standard (Tables 2 and 3, Figs 4A and 5B). Although body torso mapping of TWA is not practical for population screening, our findings highlight the potential for TWA signal underestimation using standard clinical leads in some patients. This may be potentially avoided by expanding precordial sampling in patients with negative TWA tests using the standard V1-V6 ECG leads moved to adjacent chest locations [9]. Despite the well established dependence of action potential alternans on heart rate, a clinically +TWA test was not consistently maintained at higher heart rates in our study. With atrial or atrioventricular pacing at 120 bpm, 32% of patients with +TWA (V_alt_ ≥1.9uV, k ≥3) at 100 or 110 bpm (measured from clinical leads) became negative (V_alt_ <1.9uV) (Tables 4 and 5). In half of these cases, ectopic beats were observed at the onset of the rate change to 120 bpm, which nulled the alternans signal by introducing a phase change [26]. However, the remaining patients had no ectopic beats and the apparently—TWA test at 120 bpm was due to relocation of the alternans signal outside the sampling region encompassed by the clinical leads (Fig 6). Although this would not strictly change the classification of a clinically +TWA test which is measured at ≤110 bpm, these findings have implications for interpreting TWA signal loss during exercise testing or incremental pacing.

Although exercise testing is used in most patients for clinical TWA testing, up to half of heart failure patients have indeterminate test results because inadequate heart rates are achieved [27]. Therefore, atrial pacing has a role in those unable to exercise or with chronotropic incompetence [28,29]. RV pacing may be an alternative when atrial pacing is not possible due to rate-dependent AV block or atrial fibrillation, the latter being prevalent in 30% of heart failure patients. In contrast to the high concordance rate for clinical TWA test results between exercise and atrial pacing, [30] this rate is quite variable (57–83%) when atrial pacing is compared to ventricular pacing [10–14]. In the present study, 21% of patients with clinically +TWA became negative when ventricular activation was changed from intrinsic to paced (Figs 4–6). This arose from spatial redistribution of the TWA signal from the clinical leads to the subjacent body torso in half of these patients. In order to avoid potentially false-negative clinical TWA testing when ventricular pacing is deemed necessary, the utility of a larger precordial sampling area should be evaluated in future studies.

### Limitations

Our sample size is small and did not permit evaluation of the prognostic utility of TWA signal detected outside the clinical leads, including TWA_max_ and the number of unipolar leads with detectible signal. Second, TWA was evoked with pacing and not exercise, which was not technically feasible with our 114-body surface vest. Nonetheless, the concordance rate for clinical TWA testing is reported to be high between atrial pacing and exercise [30]. Third, we did not withhold beta-blockers prior to TWA testing, which is not routinely performed in clinical TWA testing. Beta-blockers can attenuate TWA magnitude; thereby potentially reducing the likelihood of a positive TWA test [31]. However, this attenuation should be consistent in the same patient between different pacing rates and pacing modes; thereby minimizing any confounding effect. Finally, we cannot infer the presence of discordant alternans from the body surface distribution of TWA or its change during increasing heart rate or varying pacing modes. In a simulation study, we previously showed that discordant alternans in a heart model did not change the body surface distribution of alternans compared to concordant alternans, but the magnitude of body surface alternans was significantly larger [9]. Therefore, high magnitude TWA on the body surface, irrespective of location may suggest discordant alternating sources in the heart.

### Conclusions

The body surface distribution of TWA is dependent on heart rate and the sequence of ventricular activation in patients with cardiomyopathy, which may cause inconsistent TWA signal detection using the standard clinical leads. As a consequence, clinical TWA test results can be “false negative” with heart rates ≤110bpm (21%), heart rates >110bpm (when positive at lower heart rates) (11%), or with ventricular pacing (when positive during atrial pacing) (21%). In half of these cases, TWA signal migration outside the recording field of the clinical leads is the cause. Increasing the precordial sampling area in patients deemed TWA negative may improve TWA signal detection and reduce “false-negatives”.

## Abbreviations

bpm: beats per minute
ICD: implantable cardioverter defibrillator
LV: left ventricle
LVEF: left ventricular ejection fraction
TWA: T wave alternans

## References

[1] Pastore JM, Girouard SD, Laurita KR, Akar FG, Rosenbaum DS. Mechanism linking T-wave alternans to the genesis of cardiac fibrillation. *Circulation* 1999;99:1385–1394. 10077525 10.1161/01.cir.99.10.1385

[2] Shusterman V, Goldberg A, London B. Upsurge in T wave alternans and nonalternating repolarization instability precedes spontaneous initiation of ventricular tachyarrhythmias in humans. *Circulation* 2006;113:2880–2887. 10.1161/CIRCULATIONAHA.105.607895 16785339

[3] Swerdlow C, Chow T, Das Mithilesh D, Gillis A, Zhou X, Abeyratne A, Ghanem R. Intracardiac electrogram T wave alternans/variability increases before spontaneous ventricular tachyarrhythmias in implantable cardioverter-defibrillator patients: A prospective, multicenter study. *Circulation* 2011;123:1052–1060. 10.1161/CIRCULATIONAHA.110.986364 21357826

[4] Hohnloser S, Ikeda T, Cohen R. Evidence regarding clinical use of microvolt T wave alternans. *Heart Rhythm* 2009;6:S36–S44. 10.1016/j.hrthm.2008.10.011 19168396

[5] Chow T, Kereiakes DJ, Onufer J, Woelfel A, Gursoy S, Peterson B, Brown ML, Pu W, Benditt DG. Does microvolt T wave alternans testing predict ventricular tachyarrhythmias in patients with ischemic cardiomyopathy and prophylactic defibrillators? *J Am Coll Cardiol* 2008;52:1607–1615. 10.1016/j.jacc.2008.08.018 18992649

[6] Gold MR, Ip J, Constantini O, Poole J, McNulty S, Mark D, Lee K, Bardy G. Role of microvolt T wave alternans in assessment of arrhythmia vulnerability among patients with heart failure and systolic dysfunction: Primary results from the T wave alternans sudden cardiac death in heart failure trial substudy. *Circulation* 2008;118:2022–2028. 10.1161/CIRCULATIONAHA.107.748962 18955671 PMC2777708

[7] Bloomfield DM, Hohnloser SH, Cohen RJ. Interpretation and classification of microvolt T wave alternans tests. *J Cardiovasc Electrophysiol* 2002;13:502–512. 12030535 10.1046/j.1540-8167.2002.00502.x

[8] Verrier RL, Klingenheben T, Malik M, El-Sherif N, Exner DV, Hohnloser SH, Ikeda T, Martínez JP, Narayan SM, Nieminen T, Rosenbaum DS. Microvolt T-wave alternans physiological basis, methods of measurement, and clinical utility-consensus guideline by International Society for Holter and Noninvasive Electrocardiology. J Am Coll Cardiol 2011;58(13):1309–24. 10.1016/j.jacc.2011.06.029 21920259 PMC4111570

[9] Selvaraj R, Suszko A, Subramanian A, Sivananthan D, Hill A, Nanthakumar K, Chauhan VS. Body surface projection of action potential duration alternans: A combined clinical-modeling study with implications for improving T wave alternans detection. *Heart Rhythm* 2009;6:1211–1219. 10.1016/j.hrthm.2009.04.002 19632636

[10] Raatikainen MJ, Jokinen V, Virtanen V, Hartikainen J, Hedman A, Huikuri HV. Microvolt T wave alternans during exercise and pacing in patients with acute myocardial infarction. *Pacing Clin Electrophysiol* 2005;28:S193–S197. 10.1111/j.1540-8159.2005.00110.x 15683495

[11] Shalaby AA, Voigt A, El-Saed A, Mains M, Shusterman V. Microvolt T wave alternans during atrial and ventricular pacing. *Pacing Clin Electrophysiol* 2007;30:S178–S182. 10.1111/j.1540-8159.2007.00633.x 17302700

[12] Anh D, Srivatsa U, Bui HM, Vasconcellos S, Narayan SM. Biventricular pacing attenuates T wave alternans and T wave amplitude compared to other pacing modes. *Pacing Clin Electrophysiol* 2008;31:714–721. 10.1111/j.1540-8159.2008.01074.x 18507544

[13] Ehrlich JR, Wegener FT, Anneken L, Duray G, Israel CW, Hohnloser SH. Biventricular pacing does not affect microvolt T wave alternans in heart failure patients. *Heart Rhythm* 2008;5:348–352. 10.1016/j.hrthm.2007.10.032 18313590

[14] Kraaier K, Verhorst PM, Van Der Palen J, Van Dessel PF, Wilde AA, Scholten MF. Microvolt T wave alternans during exercise and pacing are not comparable. Europace 2009;11:1375–1380. 10.1093/europace/eup253 19758980

[15] Frank E. An accurate, clinically practical system for spatial vectorcardiography. *Circulation* 1956;13:737–749. 13356432 10.1161/01.cir.13.5.737

[16] Verrier RL, Nearing BD, Kwaku KF. Noninvasive sudden death risk stratification by ambulatory ECG-based T wave alternans analysis: Evidence and methodological guidelines. *Ann Noninvasive Electrocardiol* 2005;10:110–120. 10.1111/j.1542-474X.2005.10103.x 15649246 PMC6931922

[17] Mironov S, Jalife J, Tolkacheva EG. Role of conduction velocity restitution and short-term memory in the development of action potential duration alternans in isolated rabbit hearts. *Circulation* 2008;118:17–25. 10.1161/CIRCULATIONAHA.107.737254 18559701 PMC2574713

[18] Choi B, Jang W, Salama G. Spatially discordant voltage alternans cause wavebreaks in ventricular fibrillation. *Heart Rhythm* 2007;4:1057–1068. 10.1016/j.hrthm.2007.03.037 17675081 PMC2137164

[19] Wilson L, Jeyaraj D, Wan X, Hoeker G, Said T, Gittinger M, Laurita K, Rosenbaum DS. Heart failure enhances susceptibility to arrhythmogenic cardiac alternans. *Heart Rhythm* 2009;6:251–259. 10.1016/j.hrthm.2008.11.008 19187920 PMC2764250

[20] Selvaraj R, Picton P, Nanthakumar K, Mak S, Chauhan VS. Endocardial and epicardial repolarization alternans in human cardiomyopathy: Evidence for spatiotemporal heterogeneity and correlation with body surface T wave alternans. *J Am Coll Cardiol* 2007;49:338–46. 10.1016/j.jacc.2006.08.056 17239715

[21] Sato D, Shiferaw Y, Garfinkel A, Weiss J, Qu Z, Karma A. Spatially discordant alternans in cardiac tissue: Role of calcium cycling. *Circ Res* 2006;99:520–527. 10.1161/01.RES.0000240542.03986.e7 16902177

[22] Aistrup G, Kelly J, Kapur S, Kowalczhk M, Sysman-Wolpin I, Kadish A, Wasserstrom J. Pacing-induced heterogeneities in intracellular Ca2+ signalling, cardiac alternans, and ventricular arrhythmias in intact rat heart. *Circ Res* 2006;99:65–73. 10.1161/01.RES.0000244087.36230.bf 16960102

[23] Krogh-Madsen T, Christini DJ. Action potential duration dispersion and alternans in simulated heterogeneous cardiac tissue with a structural barrier. *Biophys J*. 2007;92:1138–1149. 10.1529/biophysj.106.090845 17114216 PMC1783878

[24] Engelman Z, Trew M, Smaill B. Structural heterogeneity alone is a sufficient substrate for dynamic instability and altered restitution. *Circ Arrhythm Electrophysiol*. 2010;3:195–203. 10.1161/CIRCEP.109.890459 20133934

[25] Klingenheben T, Ptaszynski P, Hohnloser SH. Quantitative assessment of microvolt T wave alternans in patients with congestive heart failure. *J Cardiovasc Electrophysiol* 2005;16:620–624. 10.1111/j.1540-8167.2005.40708.x 15946361

[26] Narayan SM, Lindsay BD, Smith JM. Demonstration of the proarrhythmic preconditioning of single premature extrastimuli by use of the magnitude, phase, and distribution of repolarization alternans. *Circulation* 1999;100:1887–1893. 10545433 10.1161/01.cir.100.18.1887

[27] Jackson CE, Myles RC, Tsorlalis IK, Dalzell JR, Spooner RJ, Rodgers JR, Bezlyak V, Greenlaw N, Ford I, Cobbe SM, Petrie MC, McMurray JJ. Profile of microvolt T-wave alternans testing in 1003 patients hospitalized with heart failure. Eur J Heart Fail 2012;14(4):377–86. 10.1093/eurjhf/hfs010 22334727

[28] Tanno K, Ryu S, Wantanabe N, et al Microvolt T wave alternans as a predictor of ventricular tachyarrhythmias: A prospective study using atrial pacing. *Circulation* 2004;109:1854–1858. 10.1161/01.CIR.0000124717.77777.EC 15066948

[29] Cantillon DJ, Stein KM, Markowitz SM, Mittal S, Shah BK, Morin DP, Zacks ES, Janik M, Ageno S, Mauer AC, Lerman BB, Iwai S. Predictive value of microvolt T wave alternans in patients with left ventricular dysfunction. *J Am Coll Cardiol* 2007;50:166–173. 10.1016/j.jacc.2007.02.069 17616302

[30] Hohnloser SH, Klingenheben T, Zabel M, Li YG, Albrecht P, Cohen RJ. T wave alternans during exercise and atrial pacing in humans. *J Cardiovasc Electrophysiol* 1997;8:887–893. 9300295 10.1111/j.1540-8167.1997.tb00621.x

[31] Klingenheben T, Grönefeld G, Li YG, Hohnloser SH. Effect of Metoprolol and d,l-sotalol on microvolt-level T wave alternans. Results of a prospective, double-blind, randomized study. *J Am Coll Cardiol* 2001;38:2013–2019. 11738309 10.1016/s0735-1097(01)01661-8

